# Artificial Intelligence Applications in Body MRI: Opportunities and Limitations

**DOI:** 10.1002/jmri.70295

**Published:** 2026-03-13

**Authors:** Haresh Naringrekar, Abdullah Alturki

**Affiliations:** ^1^ Thomas Jefferson University Hospitals Philadelphia Pennsylvania USA

**Keywords:** artificial intelligence, body MRI, deep learning, MRCP

Artificial intelligence (AI) and machine learning concepts have long been explored in radiology, with deep learning accelerating progress in the past decade [1]. Improvements in graphics processing units (GPU), the core hardware component for AI and software developments, have led to significant innovation in this field, including pulmonary nodule detection, tumor segmentation, and pulmonary embolus detection to name a few [2, 3]. This technology has also allowed for improvements in workflow efficiency, decreasing scan times and improving integration of data from various imaging studies and the electronic medical records of patients to improve diagnostic accuracy and throughput [4]. This comes at a critical time for radiologists, where a marked increase in imaging compounded by fewer available radiologists and technologists has led to many practices being unable to keep up with the demand for imaging studies being performed in ultrasound, computed tomography (CT), and magnetic resonance imaging (MRI) [2].

AI usage in the improvement of image quality in MRI has shown promise, using end to end supervised, end to end unsupervised and generative modeling techniques for image optimization [5]. Through the use of convolutional neural networks (CNN) and U‐net deep learning‐based segmentation architecture, AI has demonstrated the capability to maintain high quality imaging with fewer data points compared to conventional reconstruction techniques. This can reduce scan time, an important factor when dealing with patient motion, and increase signal to noise, improving diagnostic accuracy. Deep learning architecture has also been used in MRI to generate high field strength imaging from low field strength inputs, potentially increasing access for advanced MRI imaging techniques to places they cannot afford 1.5 or 3 T strength MRI magnets [6]. Deep learning U‐net techniques have also helped with segmentation of anatomy and tumors, with increased automation compared to other methods with training these models though still requiring human supervision [5].

AI–based post‐processing continues to expand the capabilities of body MRI, offering different approaches to improve visualization of pathology and streamline image interpretation. Among body MRI applications, magnetic resonance cholangiopancreatography (MRCP) provides a particularly illustrative example. Emerging deep learning–driven segmentation techniques illustrate the potential of AI in MRCP; for example, to refine anatomic conspicuity by reducing signal overlap and enhancing the depiction of complex ductal structures, as quality of these sequences can be affected by surrounding intervening T2 hyperintense structures and other artifacts [7]. Comparing MRCP sequences in patients to assess for progression of disease processes such as primary sclerosing cholangitis (PSC) can be challenging as it is subjective with reader variability [8]. AI‐driven semi‐automated tools such as MRCP+ can help get objective quantitative measures, aiding in diagnosis of any potential changes in degree of biliary ductal dilation over time [4, 9]. Advances such as these reflect a broader shift toward algorithmic enhancement of existing datasets, with the goal of improving diagnostic confidence rather than fundamentally altering image acquisition.

Several important limitations/pitfalls exist with AI usage in body MRI applications. Conservative labeling approaches favor sensitivity over specificity, which can leave residual signals from nearby structures. Very faint imaging abnormalities may fall below the model's detection threshold, leading to missing pathology. The quality of AI based algorithms depends on the data set from which the algorithms are derived. Limited data sets can lead to misdiagnoses of pathology secondary to anatomic variants, age‐related changes, postoperative changes, medical devices, image artifacts, and satisfaction of search [10]. For these reasons, AI based post processed images should be considered a complement to, rather than a replacement for, conventional MRI, with careful review of source images and reconstructions to confirm findings [2]. The future integration of AI into abdominal imaging will depend not only on technical performance but also on thoughtful implementation, transparency of limitations, and continued emphasis on radiologist oversight. Ultimately, the success of these tools will be measured by their ability to support more accurate, patient‐centered care while preserving the nuanced judgment that remains central to diagnostic radiology.
